# Neoadjuvant Chemotherapy in Triple Negative Breast Cancer: Correlation between Androgen Receptor Expression and Pathological Response

**DOI:** 10.31557/APJCP.2020.21.2.563

**Published:** 2020

**Authors:** Amrallah A Mohammed, Fifi Mostafa Elsayed, Mohammed Algazar, Hayam E Rashed, Abeer Hussien Anter

**Affiliations:** 1 *Department of Medical Oncology, *; 4 *Department of General Surgery, *; 5 *Department of Pathology, Faculty of Medicine, Zagazig University, *; 3 *Department of Clinical Oncology and Nuclear Medicine, Faculty of Medicine, Suez Canal University, *; 6 *Department of Clinical Oncology and Nuclear Medicine, Mansoura University Egypt, *; 2 *Oncology Center, King Salman Armed Forces Hospital, Tabuk City, Saudi Arabia. *

**Keywords:** Quadruple negative breast cancer, triple negative breast cancer, neoadjuvant chemotherapy

## Abstract

**Background:**

There is growing evidence that the response to chemotherapy may be affected by Androgen Receptor (AR) expression suggesting that triple-negative breast cancers (TNBC) AR+ and quadruple negative breast cancer (QNBC) subtypes may have different diseases behavior.

**Methodology:**

We retrospectively estimated the predictive value of the AR expression in stage II and stage III TNBC patients treated with neoadjuvant chemotherapy (NAC) and correlated with the rate of pathological response (pCR).

**Results:**

Of 89 TNBC patients, 29 patients (32.6%) were TNBC AR+ and 60 patients (67.4) were QNBC. Most of the patients were less than 60 years old. Of note, approximately 62% in the QNBC group were less than 40 years old compared with 39 % in the TNBC AR+ group. The Ki-67 expression was higher in the QNBC in comparison with TNBC AR+ being 86.7% and 65.5%, respectively. QNBC subgroup showed higher rates of pCR compared with TNBC; 60% and 24%, respectively. Higher Ki-67 expression, higher grade, and lymph node involvement were statistically significantly correlated with the rate of pCR in the QNBC group (p=0.02, p=0.04, and p=0.03, respectively). In contrast, no significant association was observed between pCR and clinical-pathological features in the TNBC AR+ group.

**Conclusion:**

Our results suggested that the AR expression in TNBC may be applied as a predictive marker for NAC. TNBC AR+ had a lower rate of pCR compared with QNBC, suggesting that this subtype may have a partial chemoresistance.

## Introduction

Triple-negative breast cancer (TNBC) is a heterogeneous disease on the pathological, molecular, and clinical levels. It represents approximately 17% of all breast cancers (BCs) and have aggressive behavior compared to other molecular subtypes . QNBC is TNBC that lacks the expression of androgen receptor (AR), Estrogen receptor (ER), progesterone receptor (PR), and human epidermal growth factor receptor 2 (HER-2) and accounts for 63%-87% of TNBC with more aggressive behavior than the other molecular subtypes (Traina et al., 2018; Sutton et al., 2012; McNamara et al.,2013; Thike et al., 2014; Anand et al., 2017; Mina et al., 2017). There is increasing data that the growth of TNBC AR+ is believed to be driven via signaling through the AR (Safarpour et al., 2014 and Collins et al., 2011).

Although TNBCs are heterogeneous diseases, they are treated in the same manner. Through Gene Expression Profile (GEP), multiple subtypes had been placed with many clinical trials aimed to target different TNBC subtypes. 

The role of androgen in normal breast tissue is well established and many data in the literature about the association with BC initiation and progression. However, its predictive and prognostic role is still in need of more clarification.

Neoadjuvant chemotherapy (NAC) is the standard of care for BC patients with locally advanced or even in with early stages (Mougalian et al., 2015). 

It is established that the higher response rate for chemotherapy in TNBC did not translate into an improvement in survival outcomes. Nevertheless, many studies had suggested that achieving pCR is an effective surrogate marker for predicting long term survival outcomes (Telli et al., 2016).

The aim of the present study is to evaluate the predictive value of the AR expression in subset of patients with TNBC treated with NAC and the correlation with the rate of pCR.

## Materials and Methods

*Patients and methods*

*Eligibility criteria and sample size*

The current multi-center retrospective study included 95 patients with stage II and stage III TNBC who diagnosed and treated at the Medical Oncology and General Surgical Departments, Faculty of Medicine Zagazig University, Egypt, Clinical Oncology and Nuclear Medicine department, Mansoura University, Egypt, and the Oncology Center in King Abdullah Medical City, Saudi Arabia, between the period from March 2012 and December 2015.

Patients with good performance status (ECOG 0-2) and measurable diseases received 6-8 cycles of NAC whereas those with prior chemotherapy or having insufficient cardiac, hepatic, renal and/or bone marrow functions were excluded.

*Collection of the data and ethical aspect*

The data were collected through medical chart review. This is a retrospective chart review study with no informed consent was obtained. The data sheet for statistics was not containing patients’ identifiers and was connected to the data collection forms using a serial code number. The institutional review board (IRB) approved the study.

*Diagnosis, immunohistochemistry technique and staging system*

All cases were pathologically diagnosed after true cut needle biopsy. The cut-off value for AR, ER and PR positivity was ≥1%, and for Ki-67 was ≥14%. HER2 was assessed by IHC and/or fluorescence in situ hybridization (FISH). HER2 positive was scored as IHC 3+ or FISH (+).

QNBC was defined as any tumors with AR (-) ER (-), PR (-) and HER-2 (-) irrespective to the expression of basal cytokeratin and epidermal growth factor receptor (EGFR). 

The work-up was based on NCCN guideline (2012) and staged according to the Joint Committee on Cancer (AJCC), 7th edition (2010) staging system).

*Pathological response evaluation*

Complete pathological response (pCR) is defined as no invasive residual in the breast or nodes. Treatment regimen

The chemotherapy protocol was institutionally based. One day before each cycle, routine laboratory investigations in the form of complete blood count, liver and kidney functions were requested. After completion of the protocol, the patients underwent breast conservation surgery or modified radical mastectomy (MRM).

*Statistical analysis*

The descriptive statistics used for patients’ characteristics. The Chi-square test used to define the relationship between expressions of different markers p values below 0.05 was considered significant.

## Results

**Figure 1:**
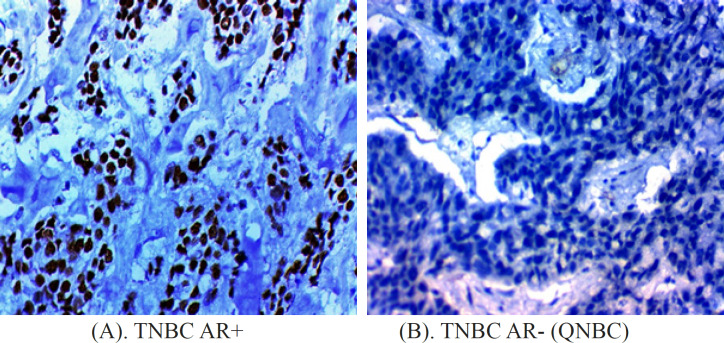
Photomicrographs of Triple Negative Breast Cancers with androgen Receptor Positive (A) and androgen receptor negative (B)Immunohistochemistry X 400 (for A and B). TNBC, triple negative breast cancer; AR+, androgen receptor positive; AR-, androgen receptor negative; QNBC, quadruple negative breast cancer

During the study period, nighty five patients diagnosed stage II-III TNBC received NAC. 6 patients were excluded due to insufficient data. Based on AR expression, 60 patients (67.4%) were AR- (QNBC), while 29 patients (32.6%) were TNBC AR+. Photomicrographs of TNBC AR+ and QNBC showed in Figure 1 (A and B) respectively. Figure 2 illustrated the treatment flow chart. 

Most of the patients were less than 60 years old. Of note, approximately 62% were less than 40 years old in QNBC group compared with 39 % in the TNBC AR+ group.

The premenopausal state represented the main bulk of our patients, being 78.3% and 55.2% of QNBC and TNBC AR+ group, respectively.

Regarding the histopathological features, there was no grade I in the studied groups. However, grade III was more common in the QNBC group, 85% versus 75.9% in the TNBC AR+.

The majority of patients in the two groups had tumor size ranged from 2 cm to 5 cm (T3 &T4); 73% and 79%, a clinical lymph node involvement in 91.7 and 100% for QNBC and TNBC AR+ respectively. By using the cutoff value 14% or more, the Ki-67 expression, the proliferative marker was higher in QNBC group compared with TNBC AR+ group (86.7% and 65.5%, respectively). Moreover, 60% of TNBC AR- patients achieved pCR compared with 24% in TNBC AR+. Clinical-pathological features and pathological response of QNBC and TNBC AR+ illustrated in Table 1. 

Higher Ki-67 expression, higher grade, and lymph node involvement were statistically significantly correlated with the rate of pCR in the QNBC group (p=0.02, p=0.04, and p=0.03, respectively). While no significant association was observed in the TNBC AR+ group. Table 2 illustrates the correlation between the pCR and different clinical-pathological features. 

Among the univariate analysis, TNBC AR-, high Ki-67 expression, IDC pathology type, and presence of LVI were associated with pCR (OR=7.960, 95% CI, p=0.001; OR=9.212, 95% CI, p=0.001; OR=0.244, 95% CI, p=0.007; OR=0.573, 95% CI, p=0.06) respectively. Table 3 illustrates a univariate analysis for predictors the pathological response.

**Table 1:** Clinical and Pathological Features of TNBC AR+ and TNBC AR

Variables	TNBC AR+		TNBC AR-		
	(No= 29)		(No = 60)		P value
	No	%	No	%	
Age (years)					
≤40	11	37.9	37	61.7	.003*
>40-60	14	48.3	22	36.7	
≥60	4	13.8	1	1.6	
Menopause status					
Premenopausal	16	55.2	47	78.3	0.1
postmenopausal	13	44.8	13	21.7	
Pathology					
IDC	25	86.2	50	83.3	0.7
Non-IDC	4	13.8	10	16.7	
Grade					
I	0	0.0	0	0.0	0.9
II	7	24.1	9	15.0	
III	22	75.9	51	85.0	
LVI					
Yes	17	58.6	24	40.0	0.6
No	12	41.4	36	60.0	
T					
T1	3	10.3	9	15.0	
T2	12	41.4	21	35.0	0.8
T3	11	37.9	23	38.3	
T4	3	10.3	7	11.7	
LN					
N0	0	0.0	5	8.3	
N1	13	44.8	22	36.7	0.9
N2	14	48.3	24	40.0	
N3	2	6.9	9	15.0	
Ki-67					
Low	10	34.5	8	13.3	.02*
High	19	65.5	52	86.7	
Pathological response					
pCR	7	24.1	36	60.0	
Non pCR					0.01*
pPR	16	55.2	20	33.3	
pSD	6	20.7	4	6.7	

TNBC, triple negative breast cancer; AR+, androgen receptor positive; AR-, androgen receptor negative; IDC, invasive duct carcinoma; LVI, lympho-vascular space invasion; T, tumor size; LN, clinical lymph node status; pCR, pathological complete response; pPR, pathological partial response; pSD, pathological stable response; *P value <0.05 signiﬁcant.

**Table 2:** The Correlation between the pCR and Different Clinical-Pathological Features

	TNBC AR+					TNBC AR-				
	No= 29					No=60				
Variables	pCR		Non pCR		P value	pCR		Non pCR		P value
	No =7		No=22			No=36		No=24		
	No	%	No	%		No	%	No	%	
Age (years)										
≤40	3	42.8	8	36.4		23	63.9	14	58.3	
>40-60	2	28.6	12	54.5	0.7	12	33.3	10	41.7	0.8
≥60	2	28.6	2	9.1		1	2.8	0	0.0	
Menopause status										
Premenopausal	5	71.4	11	50.0	0.7	22	61.1	15	62.5	0.9
postmenopausal	2	28.6	11	50.5		14	38.9	9	37.5	
Pathology										
IDC	7	100.0	18	81.8	0.6	32	88.9	18	75.0	0.2
Non-IDC	0	0.0	4	18.2		4	11.1	6	25.0	
Grade										
I	0	0.0	0	0.0	0.2	0	0.0	0	0.0	0.04*
II	2	28.6	5	22.7		7	19.4	10	41.7	
III	5	71.4	17	77.3		29	80.6	14	58.3	
LVI										
Yes	1	14.3	16	72.7	0.3	14	33.3	12	50.0	0.2
No	6	85.7	6	27.3		24	66.7	12	50.0	
T										
T1	1	14.3	2	9.1		5	13.9	5	20.8	
T2	3	42.8	9	40.9	0.7	16	44.4	12	50.0	0.3
T3	2	28.6	9	40.9		13	36.1	4	16.7	
T4	1	14.3	2	9.1		2	5.6	3	12.5	
LN										
N0	0	0.0	0	0.0		4	11.1	1	4.2	
N1	5	71.4	8	36.4	0.4	16	44.4	6	25.0	0.03*
N2	2	28.6	12	54.5		9	25.0	15	62.5	
N3	0	0.0	2	9.1		7	19.4	2	8.3	
Ki67										
Low	5	71.4	5	22.7	0.5	2	5.6	6	25.0	0.03*
High	2	28.6	17	77.3		34	94.4	18	75.0	

TNBC, triple negative breast cancer; AR+, androgen receptor positive; AR-, androgen receptor negative; IDC, invasive duct carcinoma; LVI, lymphovascular space invasion; T, tumor size; N, clinical lymph node status. *P value < 0.05 signiﬁcant.

**Table 3:** Univariate Analysis for Predictors the Pathological Response

	Pathological complete response			Non-pathological complete response			
	pCR			Non-pCR			
	OR	95%CI		OR	95%CI		P value
Variate		Lower	Upper		Lower	Upper	
Age	0.56	0.241	1.303	1.768	0.767	4.156	0.1
Menopause							
Pre vs post	0.688	0.311	1.52	1.454	0.658	3.214	0.3
Pathology							
IDC vs non IDC	0.224	0.075	0.665	4.469	1.503	13.287	0.007*
Grade							
II vs III	1.444	0.602	3.465	0.692	0.289	1.661	0.4
LVI							
Yes or No	0.573	0.215	1.039	2.114	0.963	4.641	0.06*
Tumor size	1.174	0.523	5.823	0.852	0.269	2.694	0.7
LN	0.706	0.147	3.395	1.417	0.295	6.814	0.6
Ki 67							
Low vs high	9.212	2.521	33.66	0.109	0.03	0.397	0.001*
TNBC							
AR+ vs AR-	7.96	2.492	25.427	0.126	0.039	0.401	0.001*

IDC, invasive duct carcinoma; LVI, lymphovascular space invasion; T, tumor size; LN; lymph node status; TNBC, triple negative breast cancer; AR+, androgen receptor positive; AR-, androgen receptor negative. *P value < 0.05 signiﬁcant.

## Discussion

The elegant progress of target therapies in HR+/ Her-2+ BC forced the investigators to recognize target therapies in TNBC. The introduction of AR evaluation in these subgroups of patients is really a huge step forward as it may help to determine the disease behavior (Biswas et al., 2017).

In our results, we observed that 67.4% of TNBC are QNBC. Most of the patients (61.7%) in QNBC were at the age of 40 years or younger compared with 37.9% in the TNBC AR+ group (p=0.003). This translated into the distribution of the menopausal status, as 78% in QNBC group was a premenopausal while, it was 52% in the TNBC AR+ group. 

Generally, high Ki-67 expression was significantly associated with the TNBC subtype (Ilie et al., 2018 and Elnemr et al., 2016). In the current study, TNBC AR+ has lower proliferation rates and lower histological grade compared with QNBC. This approximated to the range of previously published studies (Traina et al., 2018; Sutton et al., 2012; McNamara et al., 2013; Thike et al., 2014; Anand et al., 2017; Mina et al., 2017).

In a meta-analysis conducted on 2,826 patients with TNBC from 13 trials. Wang et al., (2016) reported that TNBC AR+ was detected in 24.4% of the whole TNBC group and was associated with postmenopausal status, low tumor grade and high risk of nodal involvement. Consistently, Gasparini et al., (2014) reported the association with QNBC and higher tumor grade. Also, Maeda et al., (2016) showed that TNBC AR+ linked with both low nuclear grade and clinical stage (p < 0.01).

Moreover, in a cohort of 203 Asian patients with TNBC, the Ki-67 index was lower in TNBC AR+ and the incidence of metastasis was high in QNBC (McNamara et al., 2013 and Sutton et al., 2012). 

Also, in our results, higher Ki-67 expression, higher grade, and lymph node involvement were statistically significantly correlated with the rate of pCR in the QNBC group (p=0.02, p=0.04, and p=0.03, respectively). This finding matched with the results of many studies evaluated the NAC in TNBC without AR evaluation (Burstein et al., 2008, Keam et al., 2011, and Elnemr et al., 2016). In contrast, no significant association was observed between pCR and clinical-pathological features in the TNBC AR+ group. These findings may reflect the probability of being two different diseases.

Furthermore, we reported that the rate of pCR to NAC was 24.1% for TNBC AR+ group compared with 60% of QNBC group. These findings matched with voluminous previous reports (Hilborn et al., 2016; Gerratana et al., 2018; Masuda et al., 2013; Asano et al., 2016; Asano et al., 2017, and ovanović et al., 2017).

Many studies had investigated the predictive value of the AR receptor in BC and in TNBC subtype after NAC. Hilborn et al reported that pCR was12.8% and 25.4% for AR-positive and AR-negative tumors, respectively (p < 0.0001). Among the TNBC group, AR expression predicted a better DFS and OS p=0.05 and p=0.03, respectively) (Hilborn et al., 2016).

Among a retrospective analysis of 130 TNBC patients treated with NAC showed a lower pCR rate for TNBC AR+ subtype (10%) in respect to BL1subtype (52%) (Gerratana et al., 2018; Masuda et al., 2013).

Results of a prospective study by Asano et al included 61 patients with TNBC reported that after NAC, the rate of pCR was statistically significantly lower and more common disease recurrence (p=0.001, p=0.008, respectively) in TNBC AR+ compared with QNBC (Asano et al., 2016). After that, the same primary investigator prospectively evaluated the pathological response from 117 TNBC Japanese patients. The results were comparable; the pCR in TNBC AR+ was less frequent than in QNBC. Similarly, Masuda et al through a retrospective study on 146 patients with TNBC, reported lower pCR in TNBC AR+ compared with QNBC (Asano et al., 2017).

In a recent randomized phase II trial to evaluate the NAC (cisplatin, paclitaxel ± everolimus) in 145 patients with TNBC showed that low expression level of AR was linked to higher pCR than higher AR levels. Moreover, the investigators demonstrated a lack of significant changes in AR levels (before, during or after NAC), suggesting that the chemotherapy did not affect *AR* expression (Ovanović et al., 2017).

Interestingly, Gong et al., (2014) reported inferior 5-year survival in patients with QNBC compared with TNBC AR+. Three meta-analyses had done by Kim et al., (2015); Wang et al., (2016) and Qu et al., (2013) reported the significant association with TNBC AR+ and favorable prognosis.

Although most of the previous studies and our results reported the possibility of the use of* AR* expression as a negative prognostic marker for TNBC, there are few trials had contradicted these results. In a large cohort included 492 patients with TNBC, the authors observed that AR+ was a poor prognostic marker for early-stage TNBC (Choi et al., 2015). 

The variations in results may be related to differences in methodology (e.g. different cut off value of AR expression of various studies) or due to variations in patients’ characteristics.

Therefore, from the mentioned above and in comparison with QNBC group, TNBC AR+ has a lower *Ki-67* expression, lower histological grade, smaller tumor size, more patients are postmenopausal, lower pCR rate after chemotherapy. Consequently, AR may be a negative predictive marker for the NAC setting. So theses subtype of patients may need more aggressive NAC or combined with an anti-androgen to improve the pathological response and subsequently reflected in survival outcome.

These findings raise the suggestion that TNBCs AR+ patients may represent a subset of patients with unique clinical-pathological features.

Till now, there are no data to support or prevent the routine use of AR assessment even through TNBC. Owing to the availability of anti-androgens such as bicalutamide, many trials had evaluated its use in the metastatic TNBC (Chae et al., 2013; Micello et al., 2010).

Furthermore, preliminary results demonstrated that *AR* expression may reduce TNBC radiosensitivity. However, there is some data about the use of bicalutamide may restore sensitivity. More evidence is needed to conﬁrm these results.

## Limitations

Retrospective studies are always criticized as the data depend totally on the medical file documentation as well as small sample size represented the primary limitation in our study.

In conclusion, TNBC AR+ is a unique subtype with distinct prognosis and clinical behavior. AR is a promising predictive marker for TNBC to NAC mainly in our country (developing country) due to the high economic poverty.

## Conflict of interest

The authors certify that there is no potential or actual conflict of interest related to this research.
